# Sclerotic Bone Metastasis in Pulmonary Adenocarcinoma

**DOI:** 10.1155/2018/1903757

**Published:** 2018-06-12

**Authors:** Riyadh Ali Mohammed Hammamy, Khalid Farooqui, Wisam Ghadban

**Affiliations:** Department of Medicine, Hamad Medical Corporation, Doha, Qatar

## Abstract

Pulmonary adenocarcinoma is one of the major types of lung cancers in which metastasis is very common and it accounts approximately to one-third of all primary pulmonary cancers. Although a minority of patients with lung cancer are asymptomatic, which gets usually detected in routine chest radiography, most of the patients present with some symptoms. Lung cancer metastasis may occur virtually in every organ system. Patients with non-small-cell lung cancer commonly have extrathoracic metastases to the adrenal glands, liver, brain, bones, and lymph nodes at presentation. Approximately one-third of patients with lung cancer will present with symptoms related to extrathoracic spread. Metastasis to the bone is not uncommon in lung cancer; however, osteoblastic bone metastasis is very rare. Here we present a 30-year-old female diagnosed to have pulmonary adenocarcinoma with multiple sclerotic bony lesions in the vertebra.

## 1. Background

Pulmonary adenocarcinoma is one of the most common types of lung cancers accounting for almost one-third of all lung cancers. Although a minority of patients with lung cancer are asymptomatic, which gets usually detected in routine chest radiography, most of the patients present with some signs or symptoms [1].

Lung cancer metastasis may occur virtually in any organ. Patients with non-small-cell lung cancer commonly have extrathoracic metastases to the adrenal glands, liver, brain, bones, and lymph nodes at presentation. However, approximately one-third of patients with lung cancer will present with symptoms related to extrathoracic spread [2].

Although metastasis to the bone is not uncommon in lung cancer, osteoblastic ones have been very rare. Miyazaki et al. described a case of pleuritis carcinomatosa and a case of small-cell carcinoma of the lung with sclerotic bone metastasis [3]. Adenocarcinoma of the lung is the most common in women and nonsmokers.

Pulmonary adenocarcinoma is usually symptomatic because of extrathoracic spread rather than primary tumor per se. Pleural metastasis also accounts for approximately 33% of extrapulmonary spread.

In a retrospective study of 259 non-small-cell lung cancer (NSCLC) patients by Tsuya et al., in about one half of the patients, the most common site of skeletal metastases was the spine [4].

Lytic bone metastases are due to a variety of primary tumors and are more common than sclerotic metastases [5]. Most of the cases mentioned in the literature till date found to have lytic bone metastasis or mixed lytic sclerotic bone metastasis with primary pulmonary adenocarcinoma.

Here we report an extremely rare case of pulmonary adenocarcinoma with sclerotic (osteoblastic) bone metastases.

## 2. Case Report

We present a 30-year-old female patient, who was admitted with low back pain and generalized body ache for 2 months prior to presentation; it was excruciating pain especially during nighttime and not much relieved by simple analgesics. The patient has a history of poor appetite with weight loss of about 4-5 kg in a span of 3 months, otherwise had no pulmonary symptoms. She is a nonsmoker and has no past medical illnesses.

Upon physical examination, the patient had bilateral discrete small cervical and axillary lymphadenopathy, and the breast examination was normal. Other systemic examination was not significant.

**Figure 1:**
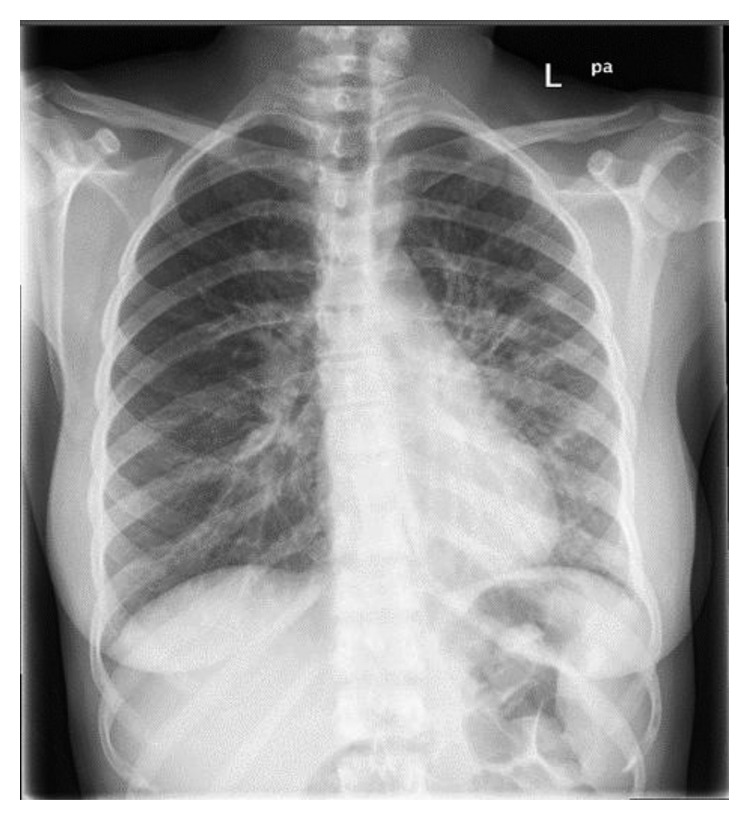
Consolidation at both perihilar regions more on the left side with possible small nodules.

**Figure 2:**
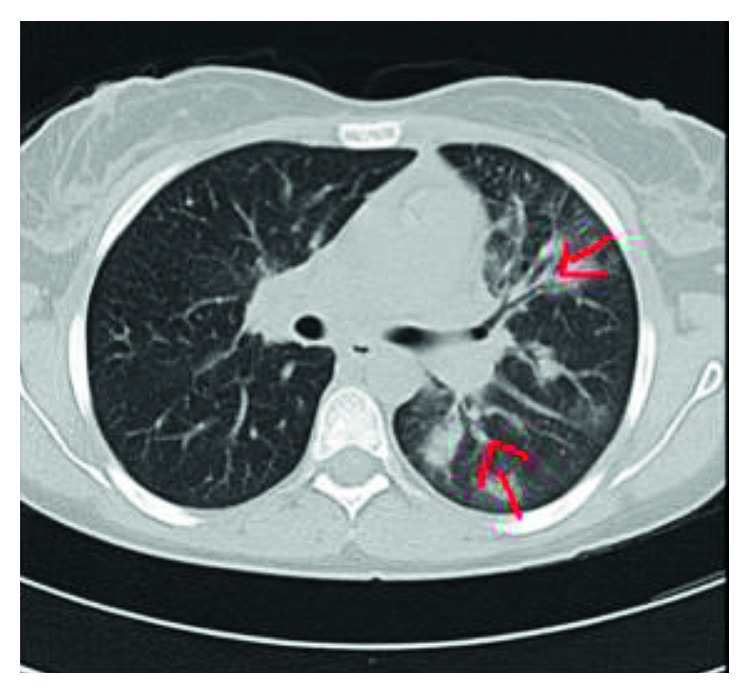
Axial lung window shows scattered areas of multifocal consolidation noted in the left lung.

**Figure 3:**
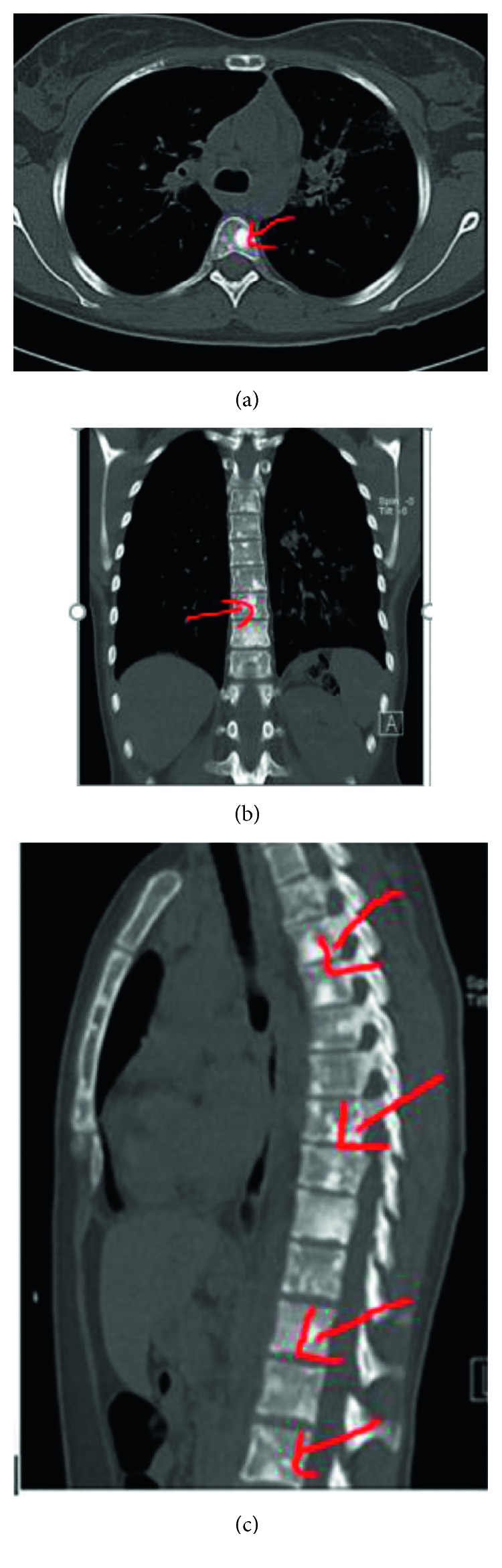
CT bone window showing sclerotic bone metastases.

During routine workup in emergency, a chest X-ray was done, which was suggestive of bilateral fluffy hilar opacities (Figure 1), and a CT thorax was done (Figures 2 and 3), which was suggestive of scattered areas of multifocal consolidation noted in the left lung and areas of scattered mosaic perfusion noted in the subpleural region, small nodules are also noted in the right lung, and both hila are prominent. The bone window shows multiple sclerotic bony lesions in the vertebra of variable sizes. There is no evidence of any collapse of the vertebra. The spinal canal diameter is normal. No spinal canal stenosis was seen.

Blood investigation showed normal CBC, electrolytes, urea, creatinine, and calcium, ESR was elevated 50 mm/hr, and high alkaline phosphatase (ALK) (224 U/L; normal 40–150 U/L), and other bone tumor markers were not done as not available.

Ultrasound (US) neck showed bilateral cervical lymphadenopathy; right-side nodes are noted, the largest of which measures 21 × 10 mm in size. Left-side nodes are noted, the largest of which measures 12 × 9 mm in size, and US breast examination was normal.

**Figure 4:**
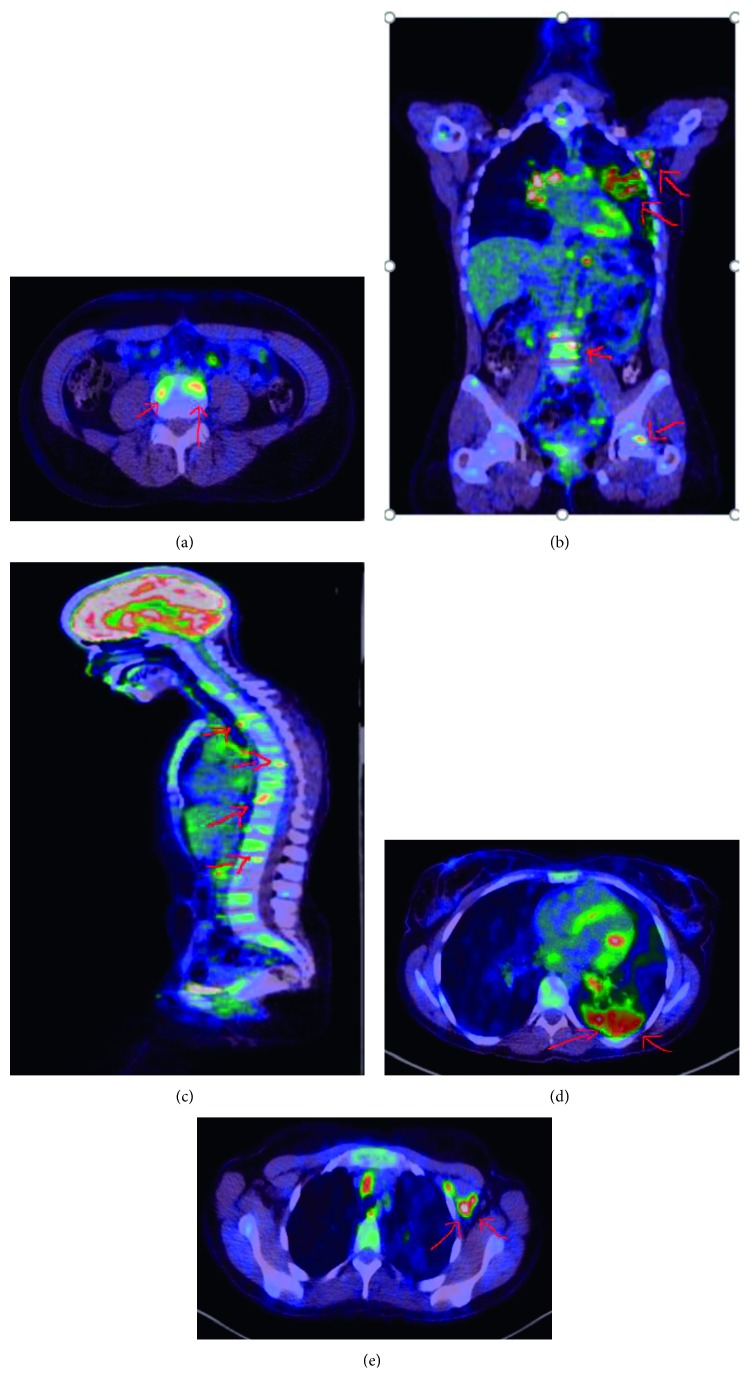
PET scan before treatment: (a) bone metastasis (hot lesions); (b) left axillary lymph nodes, and bone and lung metastasis; (c) multiple spine metastasis; (d) left lung hot lesion (adenocarcinoma); (e) left axillary lymph node metastasis.

Whole-body PET scan (Figure 4) showed progressing pulmonary consolidations and nodules compared to the CT, multiple osseous involvements, generalized, metabolically active lymphadenopathy involving supra- and infradiaphragmatic regions, and hepatomegaly with solitary hepatic lesion.

Axillary lymph node biopsy was done, and the histopathology finding is most consistent with metastatic lung adenocarcinoma. The immunohistochemical (IHC) profile is most consistent with lung primary. Breast, ovary, and biliary tract are excluded by additional IHC with appropriate controls, which is positive for Napsin A and is negative for GATA-3.

**Figure 5:**
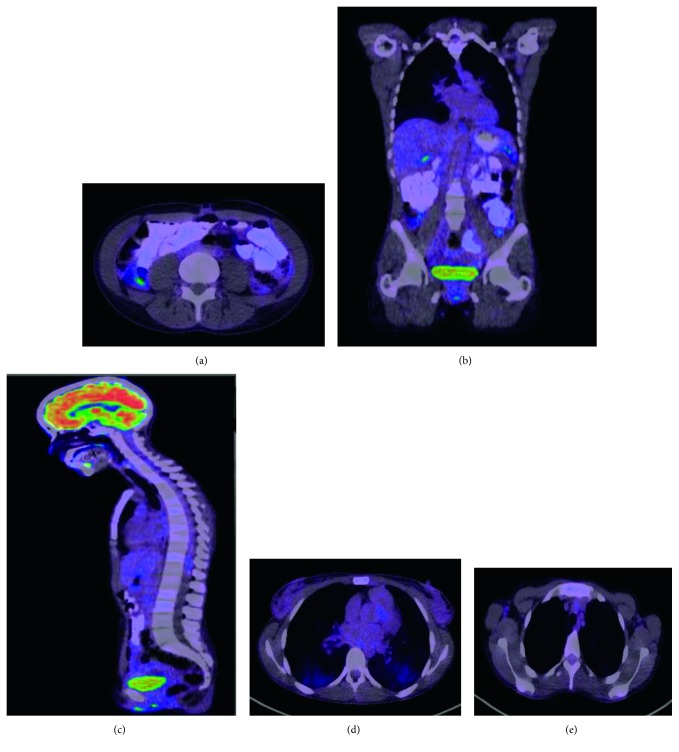
PET scan after treatment: (a) no more hot lesions in the bone; (b) no more hot lesions in the axilla, bone, and lung; (c) no spine metastasis; (d) no hot lesion in the lung; (e) no hot lesion in the left axillary lymph node.

Patient was referred to Cancer Centre for further plan of management. Patient started on chemotherapy with crizotinib 200 mg BID and denosumab 120 mg·q 4 weekly subcutaneous with good response according to the new PET scan (Figure 5). As compared to the previous PET scan 7 months before, there is favorable treatment response, metabolic resolution of previously seen left lung uptakes and lymph nodes above and below the diaphragm and metabolic resolution of bone metastases with increasing sclerosis also denoting favorable response.

## 3. Discussion

Pulmonary adenocarcinoma is one of the major types of lung cancers in which metastasis is not uncommon. About 30 to 40 percent of people with advanced lung cancer have bone metastasis. In fact, as survival rates for lung cancer are increasing, the number of people living with bone metastases is also increasing [2, 3, 6].

Bone metastatic lesions is presented by the existence of osteolytic (bone resorbing) and osteoblastic (bone forming) tumors. A third category of lesions is clinically evident in which a mixture of the two phenotypes is seen [7].

Osteolytic metastases are a consequence of tumor-induced activation of bone matrix resorption. Resorption of mineralized bone matrix is the natural function of osteoclasts, a multinucleated cell of hematopoietic origin residing in the bone. It has also been shown that interleukin (IL)-8 and chemokine ligand 2 (CCL2) can induce precursors to undergo osteoclastogenesis through a receptor activator of NF-kappaB ligand (RANKL)/RANK-independent mechanism. Osteoblastic metastases are common in advanced prostate and breast cancer patients and induced by cancer cell interactions with osteoblasts and their progenitors by production of transforming growth factor *β* (TGF-*β*), bone morphogenetic protein, insulin-like growth factor (IGF), fibroblast growth factor (FGF), and WNTs [8–10].

Osteoblastic metastases share a similar pathophysiology with osteolytic metastases, in which the bone microenvironment enhances the local growth of tumor cells, and tumor cells localized to bone in turn secrete factors that stimulate osteoblast activity and bone formation [11].

Specifically, the production of prostaglandins, parathyroid hormone, parathyroid hormone-related peptide, activated vitamin D, IL-6, and TNF by cancer cells may lead to tumor-induced increases in RANKL expression on osteoblasts and bone marrow stromal cells [7].

Sclerotic or blastic bone metastases can arise from a number of different primary malignancies including prostate carcinoma (most common), breast carcinoma (may be mixed), transitional cell carcinoma (TCC), carcinoid, medulloblastoma, neuroblastoma, mucinous adenocarcinoma of the gastrointestinal tract (e.g., colon carcinoma, gastric carcinoma), and lymphoma [5, 12, 13].

Commonly lytic bone metastases arise from lung cancer, and sometimes mixed sclerotic and lytic metastases can be seen. In our case, young aged women with no pulmonary symptoms was investigated for back pain and weight loss and was found to have pulmonary adenocarcinoma with extensive purely sclerotic bone metastases involving the entire spine and pelvis. This is one of the rare presentations of pulmonary adenocarcinoma with sclerotic bony metastases, and no cases have been reported as of our knowledge.

In conclusion, any sclerotic bone metastases should also raise high index of suspicion for the uncommon type of malignancy such as pulmonary adenocarcinoma other than the common one when diagnosis is in dilemma, and PET scan can be helpful to localize accessible tissue for biopsy.
